# Recent advances in double-lumen tube malposition in thoracic surgery: A bibliometric analysis and narrative literature review

**DOI:** 10.3389/fmed.2022.1071254

**Published:** 2022-12-14

**Authors:** Xi Zhang, Dong-Xu Wang, Jing-Qiu Wei, He Liu, Si-Ping Hu

**Affiliations:** ^1^Department of Anesthesiology, Huzhou Central Hospital, The Affiliated Huzhou Hospital, Zhejiang University School of Medicine, Affiliated Central Hospital of Huzhou University, Huzhou, China; ^2^Department of Anesthesiology, The First Affiliated Hospital, Zhejiang University School of Medicine, Huzhou, China; ^3^Department of Education and Training, Huzhou Central Hospital, The Affiliated Huzhou Hospital, Zhejiang University School of Medicine, Affiliated Central Hospital of Huzhou University, Huzhou, China

**Keywords:** double lumen tube, thoracic surgery, malposition, one-lung ventilation, airway management, fiberoptic bronchoscopy, bibliometric analysis

## Abstract

Thoracic surgery has increased drastically in recent years, especially in light of the severe outbreak of the 2019 novel coronavirus disease (COVID-19). Routine “passive” chest computed tomography (CT) screening of inpatients detects some pulmonary diseases requiring thoracic surgeries timely. As an essential device for thoracic anesthesia, the double-lumen tube (DLT) is particularly important for anesthesia and surgery. With the continuous upgrading of the DLTs and the widespread use of fiberoptic bronchoscopy (FOB), the position of DLT in thoracic surgery is gradually becoming more stable and easier to observe or adjust. However, DLT malposition still occurs during transferring patients from a supine to the lateral position in thoracic surgery, which leads to lung isolation failure and hypoxemia during one-lung ventilation (OLV). Recently, some innovative DLTs or improved intervention methods have shown good results in reducing the incidence of DLT malposition. This review aims to summarize the recent studies of the incidence of left-sided DLT malposition, the reasons and effects of malposition, and summarize current methods for reducing DLT malposition and prospects for possible approaches. Meanwhile, we use bibliometric analysis to summarize the research trends and hot spots of the DLT research.

## Introduction

Lung isolation and one-lung ventilation (OLV) are major components of anesthesiologic management for modern thoracic anesthesia and surgery (1), and the landmark invention of the double-lumen tube (DLT) allows for successful lung isolation and OLV under DLT intubation (1–3). The DLT can effectively isolate the ventilation pathways of both lungs to ventilate separately at the bronchial level, result in full deflation of the operated lung and good exposure to the operative field to facilitate surgical operations, and also prevent pus, bronchial secretions, or blood from the operated lung entering the healthy lung (1). These important functions may be disrupted once the DLT position is incorrect, so it is crucial to ensure the accurate position of the DLT.

With the innovative modification of some DLT types, continuous visualization monitoring and recording of the DLT position can contribute significantly to the reduction of DLT malposition. Fiberoptic bronchoscopy (FOB) is the most effective and reliable method to observe the DLT position (2, 4–7), and Campos urged that the DLT position would be confirmed by a FOB both in the supine and lateral positions (3). The FOB enables visualization of the DLT position, allowing timely detection and correction of DLT malposition. However, there are still risks associated with the multiple uses of FOBs. The classical standard of the correct DLT position is defined by the FOB as follows: a clear view into the left upper and lower lobe bronchus through the endobronchial lumen with the bronchial cuff directly beneath the carina and the main left bronchus should be just visible through the tracheal lumen (4). Now, with the use of the fifth-generation Broncho-Cath, the optimal position is that the ring mark placed between the tracheal cuff and bronchial cuff is just located at the tracheal carina except for patients whose height was lower than 150 cm. Considering that the left-sided DLT is the optimum choice in thoracic surgery (3), this review only discusses the placement of DLT in the left bronchial lumen. This is mainly due to its reliability and higher “margin of safety,” defined by Benumof et al. (8) as the length of a tracheobronchial tree over which it may be moved or positioned without obstructing the airway. To comprehend the research trends and hot spots of the DLT, we also conducted a bibliometric analysis by the software CiteSpace using mathematical and statistical methods to get the quantitative analysis of publications, which will assist researchers in quickly identifying current trends in the field of DLT studies (9).

## The bibliometric analysis of publications in DLT research

A bibliometric analysis of all publications in DLT research from 1950 to the present was performed using CiteSpace (vision 6.1.R2), and all publications were obtained from the Web of Science core Collection (WOSCC), and at last, a total of 1,172 publications were used for analysis after filtering out letters and removing reprints.

**Figure 1:**
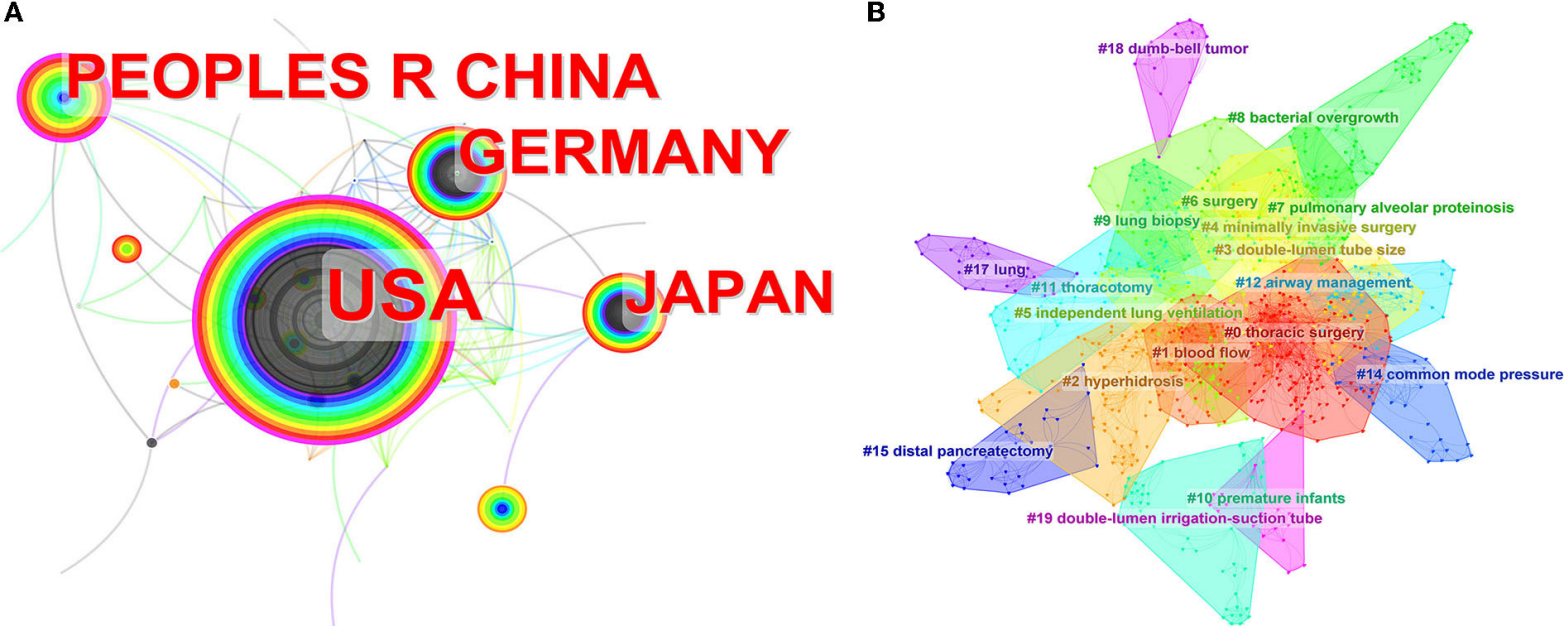
**(A)** The network map of collaborating countries in DLT research. Annular size, reflecting the number of publications; purple circle size, reflecting the intensity of centrality (generally considered to be meaningful when >0.1), and centrality is reflecting the degree to which a node is connected to other nodes throughout the network. **(B)** The clustered network map of keywords on DLT research.

The network visualization of countries in Figure 1A shows that since 1950 to date, the United States has been the country that publishes most of the papers (281), followed by China (121) and Germany (111). In contrast to developed countries such as the United States and Germany, the first article on DLT from China was published in 1996. Since then, the number of publications has been catching up with Germany, and it is not difficult to infer that this is related to the huge population base and the recent rapid development of medical care in China. In addition, the United States has the highest centrality (0.17), indicating that the United States has a significant influence on DLT research as the center of national cooperation and exchange with other countries. It is worth noting that although the research in China started late, the centrality in China is a staggering 0.14.

From the keyword clustering analysis shown in Figure 1B, the co-occurrence of the keywords suggested 19 clusters that represent the hot topics. It can be seen that the current research of scholars on DLT mainly focuses on thoracic surgery, DLT size, airway management, and independent lung ventilation. However, there is a paucity of studies on DLT malposition.

## The definitions of DLT malposition

Different studies have different definitions of DLT malposition; it can be defined as tube position, that if not corrected, might lead to clinical problems, which is defined by Campos et al. (10) as a bronchial cuff more than halfway out or not visible in the entrance of mainstem bronchus, or in the opposite bronchus, or unable to distinguish tracheal/bronchial anatomy. Malposition also can be defined as a deviation from the optimal placement to provide an easy quantitative measure, and the deviation mainly focused on 0.5 or 1.0 cm (6, 11–14).

Specifically, Inoue et al. (11) defined DLT malposition as the tube which must be pushed in or out more than 1.0 cm to correct its position and since the authors indicated that Japanese patients were small, a 1.0 cm deviation might be critical. Furthermore, Klein et al. (6) considered that the DLT malposition occurred when it had to move more than 0.5 cm to correct its position, and severe malposition was defined as the inability to see the left upper/lower lobe bronchus or intratracheal dislocation of more than one half of the endobronchial cuff. In my opinion, it may be more reasonable to set the deviation of DLT malposition at 1.0 cm, as Desiderio et al. (15) reported that DLT moved about 1.0 cm in 40 of 50 patients after lateral positioning.

## The recent incidence of DLT malposition

According to the report of studies, the DLT malposition rates vary greatly, which may be related to the differences in specifications of DLTs, definitions of malposition, and intervention measures. The incidence of left-sided DLT malposition in relevant studies over the last 20 years is shown in Table 1.

**Table 1:** The rates of left-sided DLT malposition.

**References**	**Specification of DLT**	**Definition of malposition**	**Malposition number/total**	**Rate (%)**
Campos et al. (10)	Broncho-Cath	Bronchial cuff more than halfway out or not visible in the entrance of mainstem bronchus, or in opposite bronchus, or unable to distinguish tracheal/bronchial anatomy	8/22	36.4
Inoue et al. (11)	Broncho-Cath	1.0 cm deviation from an optimal placement	49/151	32.5
Kwon et al. (12)	Broncho-Cath	1.0 cm deviation from an optimal placement	17/48	35.4
	Human Broncho	1.0 cm deviation from an optimal placement	18/52	34.6
Seo et al. (13)	Broncho-Cath	1.0 cm deviation from an optimal placement	32/50 (with pillow) 14/50 (no pillow)	64.0 28.0
Klein et al. (6)	Broncho-Cath	0.5 cm deviation from an optimal placement	80/163	49.1
Yoon et al. (14)	Broncho-Cath	0.5 cm deviation from an optimal placement	6/50 (wear neck brace) 24/50 (no neck brace)	12.0 48.0
Suzuki et al. (31)	Broncho-Cath	3-point malposition scale: 0, the tube looked to be in the same position as in the previous examination; 1, the tube was not in exactly the same position, but the bronchial lumen was not malpositioned; and 2, the cuff looked as if it was going to become herniated or dislodged.	13/26 (cuff with air^*^) 6/26 (cuff with saline ^**^)	50.0 23.1
Onifade et al. (29)	Broncho-Cath	The authors not explained	12/25	48.0
	VivaSight DLT	The authors not explained	4/25	16.0
Schuepbach et al. (27)	Broncho-Cath	The authors not explained	6/20	30.0
	VivaSight DLT	The authors not explained	5/19	26.3
Heir et al. (28)	Broncho-Cath	The authors not explained	14/42	33.3
	Vivasight DLT	The authors not explained	13/38	34.3

* Inflated with 1.2 ml of saline in the endobronchial cuff;

** inflated with 2 ml of air in endobronchial cuff. DLT, double-lumen tube.

## The reasons for DLT malposition

There are many reasons for DLT malposition, generally including shifting the patient from a supine to the lateral position, neck flexion or extension, coughing, and surgical manipulation (14, 16–18).

Desiderio et al. (15) found that 50 patients were shifted from supine to lateral position, the tracheal movement in 40 patients with an average of 0.92 cm, and the bronchial movement in 37 patients with an average of 0.92 cm. Thus, they emphasized the necessity of using a FOB to check the DLT position after lateral positioning. Furthermore, they also found that DLTs moved with lateral positioning regardless of endobronchial cuff inflation, ruling out such speculation that DLT was relatively fixed due to endobronchial cuff inflation. High rates of DLT malposition in patients after lateral positioning were also reported in studies by Klein et al. (6), Inoue et al. (11), and Maruyama et al. (19). Therefore, it is crucial to protect the DLT position as gently as possible during shifting the patient from a supine to the lateral position, and to reconfirm the DLT position using a FOB after lateral positioning before OLV.

## The complications of DLT malposition

About 40% of DLT-related complications are due to DLT malposition (20). If not promptly identified and remedied, malposition can lead to even life-threatening complications, including poor lung isolation, hypoxemia during OLV, postoperative hypoxia, atelectasis, high airway pressure, secretion accumulation, airway lacerations, and high infection rates (7, 11, 20–23).

Inoue et al. (11) found that patients with DLT malposition after lateral positioning were more likely to develop hypoxemia during OLV (28 of 29 patients), even after the correction of the DLT position by using a FOB. They suggested that the occurrence of hypoxemia in patients should be predicted in advance and checked the location of DLT first when hypoxemia occurred.

Araki et al. (24) noted in the study that endobronchial cuff pressure decreased significantly when the DLT malposition happened, even more sensitive than changes in pressure–volume loops or capnograms. Under-inflated endobronchial cuffs with low pressure may cause air leakage, obstructing the surgical field and interfering with the operation (25). Thus, we should recognize the effect of DLT malposition on endobronchial cuff pressure. However, the intraoperative monitoring of cuff pressure measurement is controversial, as the bronchial cuff volume should be inflated as minimally as the cuff could be sealed even if the volume and pressure would be over the normal values.

During OLV, DLT with the deep position where the total tidal volume is directed to only one lobe may result in barotrauma due to high airway pressure and excessive tidal volume (21). Siddik-Sayyid et al. (26) reported that a patient with atrial septal defect repair developed high airway resistance and inaudible breath sounds in both lung fields after left-sided DLT intubation and ventilation, which then occurred rapid-rate atrial fibrillation with hypotension. After they pulled the DLT back 2 cm, the bilateral breath sounds and oxygen saturation returned to normal, then they used a FOB to confirm the DLT position. The intraoperative diagnosis was left pneumothorax and released by pleural opening, then the atrial fibrillation converted to sinus rhythm and blood pressure returned to normal. They thought that rapid-rate atrial fibrillation with hypotension probably was a complication of the pneumothorax. The malposition of the DLT in this case, though being a cardiac surgery, resulted in left-sided pneumothorax, which likely caused rapid-rate atrial fibrillation with hypotension, reminding us that the diagnosis of pneumothorax should be taken into account during OLV despite the low incidence.

## Current intervention methods for reducing DLT malposition

DLT malposition rates have significantly reduced with the update in DLT types, the routine visual observation and positioning by using the FOB, and the innovation in intervention methods.

The VivaSight DLT (VS-DLT), a novel DLT with an integrated high-resolution imaging camera inserted at the end of the tracheal port, enables good visualization of the trachea and carina continuously to identify the DLT position without using a FOB (27–30). The VS-DLT has a video imaging device and light source at the distal end of the tracheal lumen, providing continuous monitoring and recording throughout the surgery, allowing for early identification of tube malposition. A recent randomized controlled trial (RCT) by Onifade et al. (29) found that the malposition rate in the VS-DLT group (16.0%) was significantly lower than in the conventional DLT group (48.0%), though previous studies by Schuepbach et al. (27) and Heir et al. (28) found that there was no statistical significance. However, none of those studies with the VS-DLT defined DLT malposition. With the advantages of successful intubation, less intubation time, rapid detection of malposition, and rapid repositioning, the VS-DLT is worthy of attention. Compared with conventional DLT, VS-DLT with a larger outer diameter may be more likely to cause minor airway damage in patients, such as hoarseness, sore throat, and blood in the trachea and ring, but there is no sufficient data to confirm that. Admittedly, the continuous visualization of the DLT position brings a great guarantee for the accurate position of the DLT and safety in thoracic surgery.

For patients with neck flexion or extension leading to DLT malposition, a study by Seo et al. (13) found that the incidence of DLT malposition was lower in patients who removed the headrest before lateral positioning compared to those who used the headrest all the time. They explained that patients without headrests had minimal neck flexion during lateral positioning. Furthermore, Yoon et al. (14) demonstrated that the use of a neck brace limiting head and neck motion minimized DLT malposition during the supine to the lateral position.

In addition, Suzuki et al. (31) found that the incidence of DLT malposition was reduced in the saline group (inflated with 1.2 ml of saline in the endobronchial cuff) compared with the air group (inflated with 2 ml of air in the endobronchial cuff), with a malposition rate of 23.1% (6/26) in the saline group and 50.0% (13/26) in the air group. However, this method may have large limitations as authors only discussed the patients receiving nitrous oxide, which has rarely been used recently due to its adverse effects.

## New attempts of DLTs and intubation methods

Takahashi et al. (32) pointed out that the SmartCuff can automatically and continuously maintain the pressure of two cuffs at the initial set pressure, slowing the rate of decline in tidal volume and effectively restoring initial tidal volume after DLT malposition. However, this study was performed on an artificial intubation model, not on clinical surgery, and there was no statistical significance in DLT malposition rates. Excessive cuff pressure can damage the mucous membranes of the trachea and bronchial tree (24), while air leakage from under-inflation of the endobronchial cuff can obstruct the operative field (25). Araki et al. (24) suggested that monitoring endobronchial cuff pressure can help in the early detection of subclinical malposition of DLT. Therefore, the clinical application of the SmartCuff, an automatic retention pressure control device, for anesthesia in thoracic surgery may contribute to reducing DLT malposition and avoiding the lower tidal volume due to malposition.

In addition, the new ANKOR DLT, invented by Dr. Young Jun, has an additional “carina cuff” that is situated at the point between the distal opening of the tracheal lumen and the beginning point of the bronchial cuff, to avoid further advancement by being blocked by the carina after inflating the cuff (33). It is more suitable for anesthesiologists with limited experience in lung isolation or patients with severe destruction of the lung parenchyma, massive pulmonary secretions, and hemothorax. Kim et al. (34) found in their study that during positioning DLT, the difference in depth between placement and target was <10 mm in 87 of 87 patients (100%) with ANKOR DLT intubation (83 patients <5 mm), while 40 of 84 patients (48%) with conventional DLT had a difference of <10 mm (26 patients <5 mm). They indicated that compared with traditional DLT, ANKOR DLT tended to be placed closer to the target depth in a more appropriate position, and its placement had less time-consuming and less traumatic. However, there is no study to observe and compare the malposition rate of ANKOR DLT. It remains to be considered whether ANKOR DLT helps to reduce the rate of DLT malposition or whether it is worth promoting.

It is likely to lead to DLT malposition when patients are shifted from a supine to the lateral position after DLT intubation. A recent study indicated that shifting patients to the lateral position increased endobronchial cuff pressure due to changes in gravity and the curvature or length of the left main bronchus (35). Thus, we hypothesized whether it was possible to assist patients in the surgically required lateral position before induction of anesthesia and then to intubate DLT in the lateral position after induction, which would directly avoid the possible adverse effects of lateral positioning. After reviewing related articles, fewer references to lateral DLT intubation can be found. Navarro-Martinez et al. (36) mentioned in their article that an essential and unique issue for non-intubated thoracic surgery (NIVATS) was the mastering of lateral position intubation for emergencies. Patients in NIVATS were likely to require DLT intubation, and lateral intubation was not difficult, according to their experiences. In addition, Ajimi et al. (37) reported a case of successful left-sided DLT intubation in the right lateral position for a patient with tracheal compression caused by a large mediastinal tumor. DLT intubation in the lateral position may be an effective new attempt to reduce the incidence of DLT malposition in thoracic surgery. Theoretically, the step of using the FOB again to confirm the DLT position after the lateral position can be eliminated. Next, we will design and conduct an RCT to explore the efficacy and clinical value of this intervention.

## Discussion

First, using a bibliometric analysis, we found that the United States holds significant power in DLT research and is the center of national cooperation. However, there are fewer studies on DLT malposition.

Currently, the number of thoracic surgeries continues to increase, and recent studies have shown that DLT malposition still happens in thoracic surgery, possibly due to shifting patients from supine to the lateral position, which results in failure lung isolation and hypoxemia during OLV. The renovation of the DLTs and the routine use of the FOB can provide a guarantee for thoracic anesthesia and surgery, but the occurrence of DLT malposition and the associated infection risks associated with multiple FOB use cannot be ignored. Recently, some innovative DLTs and improved intervention methods have shown good results in reducing the incidence of DLT malposition. Moreover, many new clinical attempts have not been proven to reduce DLT malposition, but given their particular advantages, performing studies on DLT malposition may yield positive results.

We will conduct an RCT for a new method that places patients in the surgically required lateral position before anesthesia induction and then intubate DLT in the lateral position after induction to explore the efficacy and clinical value of the lateral DLT intubation, which could be a breakthrough in DLT intubation methods. For anesthesiologists who have never tried lateral DLT intubation, this procedure seems awkward and difficult. However, with flexible intubation techniques and proper DLT shaping, this process is not as challenging as anticipated, according to the pilot trial. Lateral intubation can not only directly avoid DLT malposition caused by patient position conversion but also reduce the multiple uses of FOB to confirm the DLT position. Surgeons can start surgery as soon as the DLT is positioned correctly, eliminating the need to move the patient and speeding up the procedure. Enhanced recovery after surgery (ERAS) is what physicians are looking for and what patients desperately need. We expect to reduce or even avoid the occurrence of DLT malposition, which is more in line with the ERAS concept.

## Author contributions

XZ, D-XW, S-PH, HL, and J-QW: conceptualization. D-XW: software. XZ and D-XW: writing—original draft preparation. XZ, S-PH, HL, and J-QW: writing—review and editing. S-PH and HL: supervision and funding acquisition. All authors have read and agreed to the final version of the manuscript.
